# Graphitic Carbon Nitride and Nickel-Doped Graphitic Carbon Nitride for Doxycycline Removal: Adsorption Efficiency and Mechanistic Insights

**DOI:** 10.1021/acs.langmuir.5c03760

**Published:** 2025-12-08

**Authors:** Daniela Cristina Feitosa Angelo, Raíssa Santos Sousa, Rafael Meuredi Pinheiro Souza, Douglas Henrique Pereira, Gleice Botelho

**Affiliations:** † Programa de Pós-Graduação em Química, 74385 Federal University of Tocantins, 77402-970, Gurupi, Tocantins, Brazil; ‡ Department of Environmental Chemistry, Federal University of Tocantins, 77402-970, Gurupi, Tocantins, Brazil; § Department of Chemistry, Technological Institute of Aviation, Praça Marechal Eduardo Gomes, 50, Vila das Acácias, São José dos Campos, São Paulo 12228-900, Brazil

## Abstract

Adsorption is a simple yet effective technique for the removal of emerging contaminants. In this study, pure graphitic carbon nitride (g-C_3_N_4_) and nickel-doped g-C_3_N_4_ (Ni-*g*-C_3_N_4_) were synthesized via a thermal polycondensation method and evaluated as adsorbents for doxycycline removal. Structural and morphological characterization was performed using X-ray diffraction, Fourier transform infrared spectroscopy, X-ray photoelectron spectroscopy, field emission scanning electron microscopy, and transmission electron microscopy. The results confirmed the successful incorporation of Ni^2+^ into the g-C_3_N_4_ matrix, with homogeneous Ni^2+^ distribution and no secondary phases. Morphological analysis indicated that nickel doping induced only slight changes in the g-C_3_N_4_ morphology. Adsorption experiments revealed that both materials exhibited enhanced adsorption capacity at pH 9, with Ni-*g*-C_3_N_4_ achieving a significantly higher removal efficiency (53.4%) compared to g-C_3_N_4_ (14.2%). This improvement was attributed to Ni^2+^-induced positively charged regions, facilitating stronger adsorbate–adsorbent interactions. Kinetic analysis demonstrated that doxycycline adsorption onto Ni-*g*-C_3_N_4_ followed a pseudo-second-order model. Among the tested isotherm models, the Sips model provided the best fit, yielding maximum adsorption capacities of 37.889 mg·g^–1^ for g-C_3_N_4_ and 116.265 mg·g^–1^ for Ni-*g*-C_3_N_4_. Theoretical calculations corroborated experimental findings, confirming that Ni^2+^ incorporation in the g-C_3_N_4_ structure enhances adsorption capacity by facilitating strong chemical bonds between doxycycline and the Ni-*g*-C_3_N_4_ adsorbent surface.

## Graphical abstract

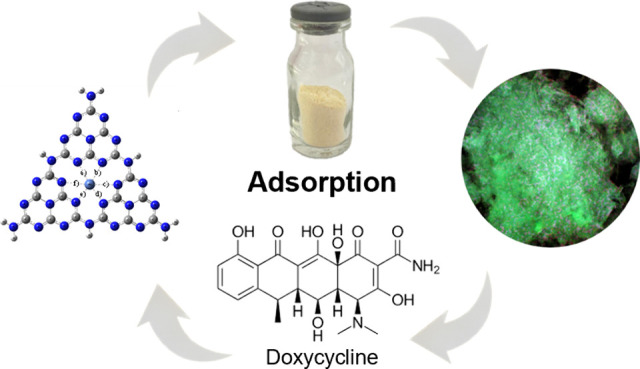

## Introduction

Pharmaceuticals are a class of chemical compounds that can alter biological metabolism and disrupt environmental dynamics when improperly disposed of, leading to various ecological impacts.^1^ These substances are widely used for treatment, prevention, and diagnosis of human and animal diseases, and are classified as emerging contaminants (ECs).^2^ They enter the environment through multiple pathways, including domestic sewage containing unmetabolized drug residues excreted in urine and feces, as well as wastewater from livestock farming.^3^

Antibiotics, in particular, are continuously released into the environment and persist even at low concentrations, posing challenges for removal and degradation. This persistence raises significant public health and environmental concerns, making antibiotic contamination a critical research focus worldwide.^1^ Among these doxycycline (Dox), a tetracycline-class antibiotic, is extensively used in human and veterinary medicine as well as in agriculture for prophylactic and therapeutic purposes.^4^ Typically, over 70% of tetracycline-class antibiotics are excreted unchanged into the environment via human and animal waste. Once released, these compounds persist in aquatic systems because of their hydrophilic nature and low volatility, which hinders their removal by conventional water treatment processes. Reported effects include toxicity in aquatic and terrestrial organisms and estrogenic and endocrine-disrupting impacts in fish and other species. In addition, their presence in the environment contributes to the development of bacterial resistance.^5^

Conventional wastewater treatment plants are largely ineffective at removing pharmaceutical contaminants, primarily due to these contaminants’ strong hydrophilicity and poor biodegradability. As a result, treated water may still contain drug residues, re-entering the residential supply. The presence of antibiotics in water sources contributes to the development of antibiotic-resistant bacteria, posing a significant public health risk.^4^ To enhance contaminant removal, advanced treatment techniques must be integrated with existing wastewater treatment processes.^6^

Among these techniques, adsorption is particularly promising due to its simplicity, high efficiency, and cost-effectiveness, making it a viable solution for water treatment.^6^ Adsorption is a separation method in which dissolved constituents in a liquid phase adhere to the surface of a solid adsorbent via physicochemical interactions, facilitating their removal from the fluid.^7^

Graphitic carbon nitride (g-C_3_N_4_) is a widely studied material known for its diverse applications, including photocatalysis,^8,9^ adsorption,^10^ and biosensing.^9^ Although g-C_3_N_4_ is a viable adsorbent, its low adsorption capacity limits its practical applications.^11^ To enhance its adsorption capacity, various modifications can be applied^12^ such as doping,^13^ hybridization with other materials,^14,15^ and morphology modulation.^10^

Metal doping is a simple and efficient method for introducing new active sites on material surfaces, thereby enhancing adsorption capacity.^12^ Recently, Meng and Nan (2022)^13^ synthesized Fe- and Na-*co*-doped g-C_3_N_4_ and evaluated the effect of doping on the adsorption of organic dyes. The doped samples exhibited significantly higher adsorption capacity than pure g-C_3_N_4_, attributed to improved electrostatic interactions with methylene blue dye. Despite the potential of metal-doped g-C_3_N_4_ as an adsorbent, few studies have explored its application in adsorption.^13,16^ To the best of our knowledge, no studies have investigated its use for the removal of pharmaceuticals via adsorption.

Metal doping of g-C_3_N_4_ generally induces changes in morphology, electronic structure, and surface properties, resulting in the formation of additional active sites and an increased surface area, which are favorable characteristics for adsorption processes.^17,18^ Several studies have reported the synthesis of Ni-doped g-C_3_N_4_; however, these studies have predominantly focused on photocatalytic applications.^19–21^ For example, Kim et al. (2021)^19^ synthesized Ni-doped g-C_3_N_4_ for the photodegradation of volatile organic compounds (VOCs) under visible light. Ni doping enhanced visible light absorption and promoted the charge separation of the electron–hole pairs, thereby enhancing VOC degradation. Moreover, the incorporation of nickel into g-C_3_N_4_ altered the morphology and increased the surface area of the doped material. Although metal-doped g-C_3_N_4_ has been extensively investigated, these investigations have been limited to photocatalytic applications.^19–24^

In addition to experimental data, theoretical calculations at the atomic and molecular levels can contribute to various experimental analyses.^25^ Computer simulations can elucidate the molecular structure and its conformations, evaluate reaction mechanisms, and compute thermodynamic, kinetic, and spectroscopic properties. Simulations can reveal key adsorption mechanisms, including the interaction energy, the spontaneity and thermal nature (endothermic or exothermic) of the process, the characteristics of the molecular interactions, and the adsorbate–adsorbent retention time.^26^ Thus, simulations contribute to the molecular analysis of the adsorption process, explaining how the interaction occurs and reducing experimental costs and time.^27^

This study aims to synthesize pure graphitic carbon nitride (g-C_3_N_4_) and nickel-doped g-C_3_N_4_ (Ni-*g*-C_3_N_4_) using a simple chemical method and to investigate their adsorption properties-both experimentally and theoretically for the removal of the pharmaceutical contaminant Dox.

## Experimental Section

### Material Synthesis

g-C_3_N_4_ was synthesized via thermal polycondensation, following a previously reported method,^12^ as shown in Scheme 1. Specifically, 10 g of melamine (C_3_H_6_N_6_, Sigma-Aldrich, 99% purity) was ground in an agate mortar, placed in a porcelain crucible, and heated in a muffle furnace at 550 °C for 3 h at a heating rate of 5 °C/min. The resulting material was allowed to cool naturally to 22 °C and then ground into a fine powder.

**Scheme 1:**
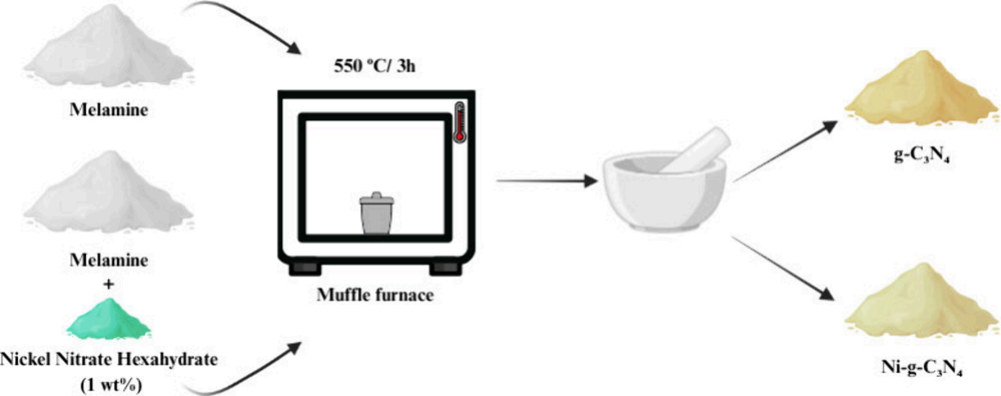
Schematic of the Synthesis of g-C_3_N_4_ and Ni-Doped Samples

Ni-*g*-C_3_N_4_ was synthesized using a modified version of the method reported by Pham et al. (2021).^19^ The synthesis followed the same steps as for pure g-C_3_N_4_ but incorporated nickel nitrate hexahydrate (Ni­(NO_3_)_2_.6H_2_O), Sigma-Aldrich, 97% purity) at a loading of 1 wt % relative to the melamine precursor. The mixture was ground and homogenized in an agate mortar, transferred to a closed crucible, and subjected to the same thermal treatment as g-C_3_N_4_.

### Material Characterization

The structural properties of both pure g-C_3_N_4_ and Ni-*g*-C_3_N_4_ were analyzed via X-ray diffraction (XRD) using a Rigaku DMax2500PC diffractometer with Cu-Kα radiation (λ = 1.5406 Å) over a 2θ range of 10°-80°. Fourier transform infrared (FTIR) spectroscopy was conducted on a PerkinElmer IR Spectrum Two spectrophotometer in the 400–4000 cm^–1^ range, with a resolution of 4 cm^–1^ and 32 accumulations. The surface chemical composition and elemental oxidation states were examined by X-ray photoelectron spectroscopy (XPS) using a Thermo Scientific K-Alpha system with monochromatic Al-kα excitation. Data were analyzed using Avantage software, and spectra were calibrated to the C 1s peak at 284.8 eV. Morphological analysis was performed using field emission scanning electron microscopy (FE-SEM) with a JEOL JSM-7100F, equipped with energy-dispersive X-ray spectroscopy. Transmission electron microscopy (TEM) and high-resolution TEM (HR-TEM) images were obtained using a JEOL JEM-2100 microscope.

### Effect of pH

The surface charges of the adsorbents was evaluated by determining the point of zero charge (pH_pzc_), following a method reported in the literature.^28^ For this, 10 mL of a 0.1-mol·L^–1^ sodium chloride (NaCl, Sigma-Aldrich, 99% purity) solution with initial pH (pH_i_) values ranging from 2 to 12 was added to 11 vials for each adsorbent, with each vial containing 10 mg of the respective adsorbent. The pH_i_ was adjusted using 0.1-mol·L^–1^ hydrochloric acid (HCl, Alfhatec, 37% purity) or sodium hydroxide (NaOH, Neon Comercial, 98.52% purity). The suspensions were stirred at 100 rpm for 24 h at a controlled temperature range of 22 °C ± 2 °C. The final pH (pH_f_) of each solution was then measured, and pH_pzc_ was determined as the arithmetic mean of the stable pH values.^28^

Adsorption experiments for g-C_3_N_4_ and Ni-*g*-C_3_N_4_ were conducted using an aqueous Dox solution (C_22_H_24_N_2_O_8_, SM Empreendimentos Farmacêuticos LTDA, 98.54% purity). Initially, the effect of pH on adsorption capacity was evaluated. For this, 25 mL of a 50 mg·L^–1^ Dox solution was placed into three flasks, and the pH was adjusted to 3, 6, and 9 using 0.1 mol·L^–1^ HCl or NaOH solutions. Subsequently, 10 mg of the respective adsorbent was added to each solution, and the mixtures were stirred at 100 rpm in an orbital shaker (MA 139/CFT/UN Marconi) at 22 °C ± 2 °C for 120 min. After adsorption, the adsorbent samples were withdrawn, centrifuged, and the supernatants were analyzed using an ultraviolet–visible (UV–Vis) spectrophotometer (T70, PG Instruments Ltd.) at a maximum wavelength (λ_max_) of 353 nm, following a methodology adapted from previous studies.^29,30^

The Dox removal percentages (%) for g-C_3_N_4_ and Ni-*g*-C_3_N_4_ were determined using eq 1:

1 $$removal\;\left(\%\right)=\frac{C_{0}-C_{e}}{C_{0}}\times 100$$

where *C*_0_ (mg·L^–1^) and *C*_e_ (mg·L^–1^) are the initial and equilibrium concentrations of Dox.

### Kinetic Studies and Adsorption Isotherms

For the kinetic studies, 10 mg of the adsorbent (g-C_3_N_4_ or Ni-*g*-C_3_N_4_) was added to vials containing 25 mL of a 50 mg·L^–1^ Dox solution, with the pH adjusted to 9. The suspensions were stirred at 100 rpm for various contact times (5, 10, 20, 30, 60, 90, 120, 180, and 360 min). Aliquots of 3 mL were then withdrawn, centrifuged, and the supernatant analyzed using a UV–Vis spectrophotometer. For the adsorption isotherm studies, 10 mg of the adsorbent was added to vials containing 25 mL of Dox solutions at different concentrations (10, 20, 30, 50, 70, 100, 150, and 200 mg·L^–1^), all with pH adjusted to 9. The suspensions were stirred at 100 rpm for 120 min, followed by centrifugation of the withdrawn aliquots, and the supernatant was analyzed using a UV–Vis spectrophotometer. All kinetic and isotherm experiments were conducted at 22 °C ± 2 °C and performed in triplicate.

The adsorption capacity (*q*) of Dox on g-C_3_N_4_ (mg·g^–1^) was calculated using eq 2:

2 $$q=\frac{C_{0}-C_{e}}{m}\times V$$

where *m* is the mass of adsorbent (g), and *V* is the volume of the Dox solution (L).

### Computer Simulations

Density functional theory calculations were performed to analyze the electronic structures of Dox, g-C_3_N_4_ and Ni-*g*-C_3_N_4_, as well as the Dox–adsorbent interactions.^31^ All structures were constructed and visualized using GaussView software,^32^ and optimization calculations were carried out in the Gaussian 09 program.^33^ Frequency calculations were performed, and no imaginary frequencies were detected, confirming the energy minimum. The M06L functional^34^ and the 6-31G­(d,p) basis set^35^ were used for carbon, oxygen, nitrogen, and hydrogen atoms, while the LANL2DZ basis set^36^ with a pseudopotential was applied for nickel. All simulations employed the implicit solvent model Solvation Model based on Density to simulate the effect of water.^37^

The frontier molecular orbitals (FMOs)—highest occupied molecular orbital (HOMO) and lowest unoccupied molecular orbital (LUMO)—and the molecular electrostatic potential (MEP) for Dox, g-C_3_N_4_ and Ni-*g*-C_3_N_4_ were generated and analyzed using GaussView software. The HOMO and LUMO energies (*E*_HOMO_ and *E*_LUMO_, respectively) were obtained and used to calculate the energy gap (*E*_GAP_), using eq 3:

3 $$E_{GAP}=E_{LUMO}-E_{HOMO}$$

Energetic and thermodynamic parameters, including interaction energy (*E*_ads_), enthalpy (ΔH), and Gibbs free energy (ΔG), were calculated for the g-C_3_N_4_–Dox and g- Ni-*g*-C_3_N_4_–Dox systems using eqs 4–6:

4 $$E_{ads}=E_{Complex}-\left(E_{adsorbent}+E_{adsorbate}\right)$$

5 $$\Delta H=H_{Complex}-\left(H_{adsorbent}+H_{adsorbate}\right)$$

6 $$\Delta G=G_{Complex}-\left(G_{adsorbent}+G_{adsorbate}\right)$$

## Results and Discussion

### Structural Characterization

The g-C_3_N_4_ and Ni-*g*-C_3_N_4_ structures were characterized via XRD, as shown in Figure 1(a). The XRD pattern of g-C_3_N_4_ displayed two distinct peaks at approximately 2θ = 13.0° and 27.6°, corresponding to the crystallographic planes (100) and (002), respectively (JCPDS No. 87-1526). These peaks are typically attributed to the interlayer packing of tri-s-triazine and the stacking of aromatic or graphitic systems, respectively.^19,22^ A similar pattern was observed for Ni-*g*-C_3_N_4_, with no additional peaks corresponding to nickel-related phases, such as nickel oxide, carbide, nitride, or Ni^0^. Additionally, no shifts in the position or reduction in the intensity of the g-C_3_N_4_ peaks were observed, indicating that the structure remained stable after nickel doping. The undisrupted crystalline structure of g-C_3_N_4_ even after nickel doping is likely due to the low concentration of the dopant used during synthesis.

**Figure 1:**
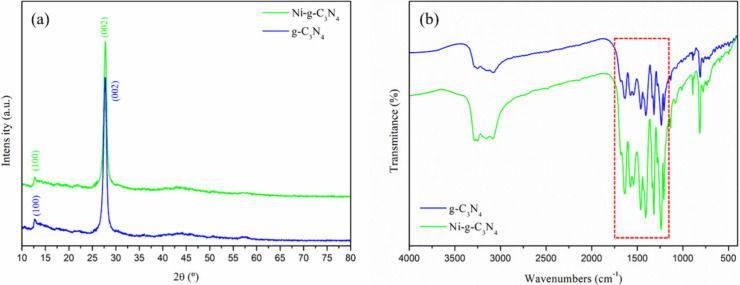
(a) XRD patterns and (b) FTIR spectra of g-C_3_N_4_ and Ni-*g*-C_3_N_4_.

FTIR spectroscopy was performed to identify the functional groups in the adsorbents based on their vibrational modes. The spectra of both g-C_3_N_4_ and Ni-*g*-C_3_N_4_ samples were similar (Figure 1(b)). A sharp peak at approximately 808 cm^–1^ corresponds to the breathing mode of tri-s-triazine units.^8,21^ The intense peaks observed between 1237 and 1638 cm^–1^ are attributed to the stretching vibrations of the aromatic rings, corresponding to C–N (1237–1459 cm^–1^) and C=N (1575–1638 cm^–1^) bonds.^8^ Additionally, the bands in the range of 3080–3249 cm^–1^ are assigned to the stretching vibrations of amine groups (N–H).^8,21^ The FTIR spectrum of Ni-g-C_3_N_4_ was similar to that of g-C_3_N_4_, with all characteristic peaks preserved, which is consistent with the XRD results. According to the study by Das et al. (2018),^38^ the samples doped with high nickel concentrations (≥3 wt %) exhibited additional peaks at approximately 2846 and 2924 cm^–1^, which were attributed to excess Ni. This is consistent with our study, where low amounts of nickel were used to preserve the structure of g-C_3_N_4_ after doping. In addition, no shifts were observed in the wavenumbers of the carbon and nitrogen bonds, indicating that Ni^2+^ doping did not alter the g-C_3_N_4_ structure.

XPS analysis was performed to evaluate the surface chemical composition and identify the chemical states of the elements present in g-C_3_N_4_ and Ni-*g*-C_3_N_4_ (Figure 2). In the survey spectrum (Figure 2(a)), both samples exhibited C 1s and N 1s peaks, along with a low-intensity peak at 532.1 eV, corresponding to O 1s. This is attributed to CO_2_ adsorption on the surface of the material.^39,40^ For the Ni-*g*-C_3_N_4_ sample, in addition to the C 1s, N 1s, and O 1s peaks, a Ni 2p peak was observed at 856.2 eV,^41^ confirming the successful doping of nickel into the g-C_3_N_4_ structure.

**Figure 2:**
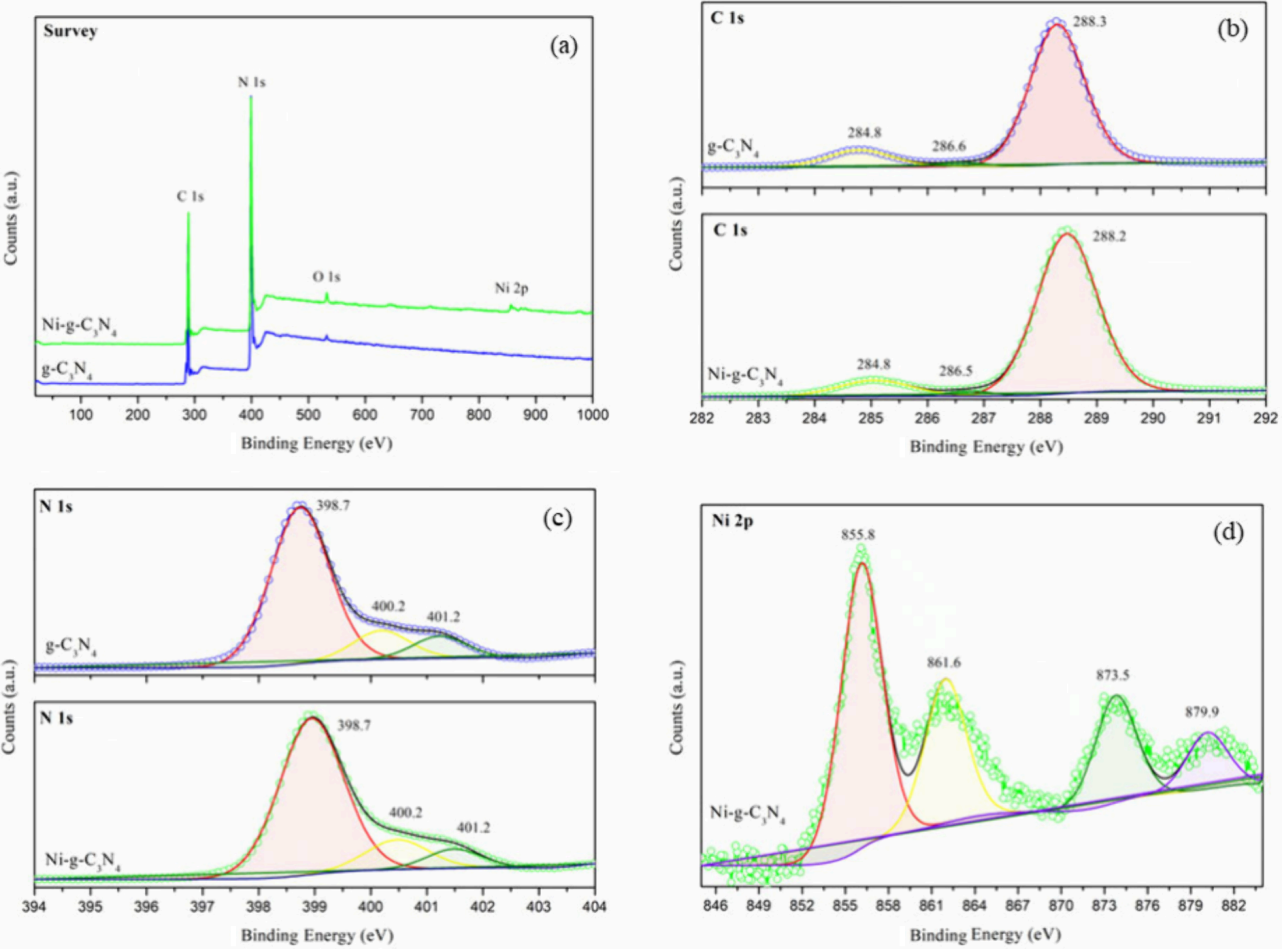
XPS spectra of g-C_3_N_4_ and Ni-*g*-C_3_N_4_: (a) survey, (b) high-resolution C 1s, and (c) high-resolution N 1s spectra of g-C_3_N_4_ and Ni-*g*-C_3_N_4_. (d) High-resolution Ni 2p spectrum of Ni-*g*-C_3_N_4_.

Figure 2(b,c) presents the high-resolution C 1s and N 1s spectra for both g-C_3_N_4_ and Ni-*g*-C_3_N_4_. In the C 1s spectrum of g-C_3_N_4_ (Figure 2(b)), three peaks were observed upon deconvolution. The main peak at 288.3 eV corresponds to sp^2^ hybridized carbon in the aromatic structure (N–C=N) of g-C_3_N_4_.^39,40^ Lower intensity peaks at 286.6 and 284.8 eV are attributed to C–O residues^40^ and graphitic carbon, respectively.^39–41^ In the N 1s spectrum of g-C_3_N_4_ (Figure 2(c)), the primary peak at 398.7 eV is associated with sp^2^ hybridized aromatic nitrogen bonded to carbon (C=N–C) in the tri-s-triazine rings.^21,39,41,42^ Additional peaks at 400.2 and 401.2 eV are attributed to nitrogen bonded to three carbon atoms in N–(C)_3_ ^21,39,41,42^ and the terminal amino functional group (C–N–H), respectively.^21,41,42^ For the Ni-*g*-C_3_N_4_ sample, similar peak patterns were observed in both the C 1s and N 1s spectra. The deconvoluted peaks were centered at 284.8, 286.5, and 288.2 eV for C 1s, and at 398.7, 400.2, and 401.2 eV for N 1s (Figure 2(b,c)). These findings suggest that nickel doping did not significantly alter the chemical environment of carbon and nitrogen in g-C_3_N_4_.

The high-resolution Ni 2p spectrum (Figure 2(d)) of Ni-*g*-C_3_N_4_ exhibits two main peaks at 855.8 and 873.5 eV, corresponding to Ni 2p_3/2_ and Ni 2p_1/2_, respectively, along with their respective satellite peaks at 861.6 and 879.9 eV.^20,41^ Notably, no peaks were observed at 852.9 eV, indicating the absence of metallic nickel (Ni^0^) formation during pyrolysis.^20,43,44^ These findings support the presence of Ni^2+^ ions and confirm the successful doping of g-C_3_N_4_ with nickel. In addition, based on the XPS results, the elemental composition of g-C_3_N_4_ was approximately 43.8 at. % C and 56.2 at. % N. For Ni-g-C_3_N_4_, the composition was approximately 43.3 atom % C, 56.1 atom % N, and 0.6 atom % Ni, confirming the successful incorporation of nickel into g-C_3_N_4_.

### Morphological Characterization

The morphology of the g-C_3_N_4_ and Ni-*g*-C_3_N_4_ samples was examined using FE-SEM microscopy (Figure 3). Figure 3(a,b) show that g-C_3_N_4_ exhibits planar, plate-like structures that are highly agglomerated, forming micrometric blocks, a morphology typical of g-C_3_N_4_ obtained via thermal polycondensation.^38,40,45^ In contrast, Ni-*g*-C_3_N_4_ (Figure 3(c,d)) displayed thinner, sheet-like structures, indicating that nickel doping influenced the morphology of g-C_3_N_4_. Elemental mapping (Figure 4) for g-C_3_N_4_ confirmed the homogeneous distribution of carbon, nitrogen, and nickel, supporting the successful doping of g-C_3_N_4_ with nickel.

**Figure 3:**
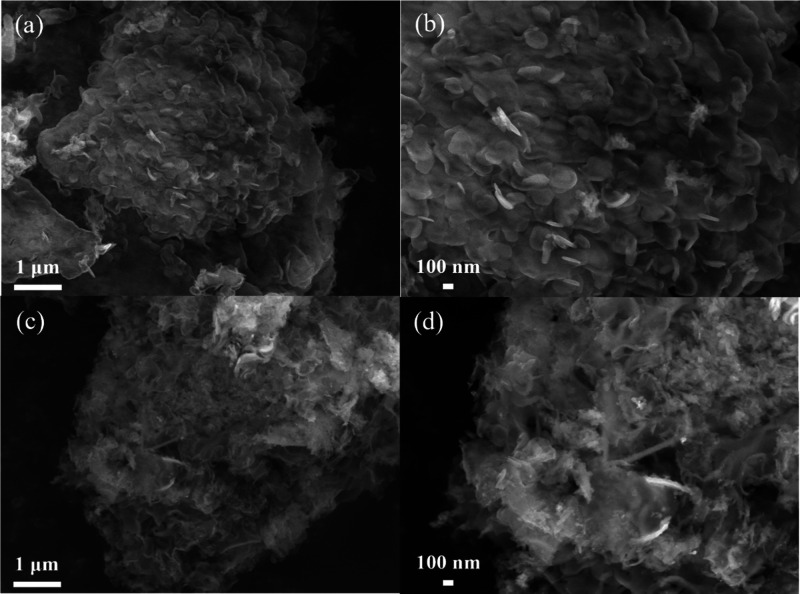
FE-SEM images of (a, b) g-C_3_N_4_ and (c, d) Ni-*g*-C_3_N_4_.

**Figure 4:**
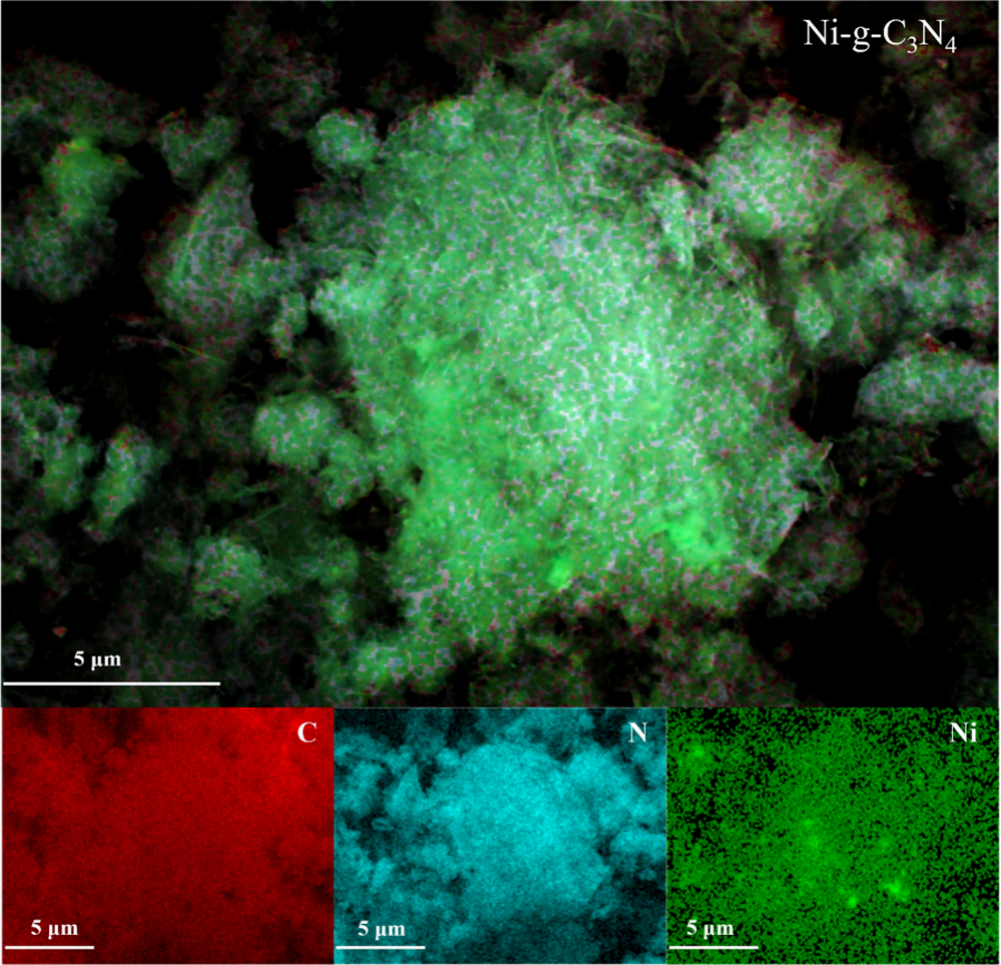
Elemental mapping of carbon, nitrogen, and nickel in Ni-*g*-C_3_N_4_.

TEM analysis was conducted to further investigate the morphology and microstructure of the obtained samples (Figure 5). The results are consistent with the FE-SEM observations, confirming that both g-C_3_N_4_ and g Ni-*g*-C_3_N_4_ are composed of planar nanostructures. TEM images of g-C_3_N_4_ (Figure 5(a,b)) and Ni-*g*-C_3_N_4_ (Figure 5(c,d)) reaffirm the trends observed in FE-SEM (Figure 3). However, in the case of Ni-*g*-C_3_N_4_, the nanostructures appear more elongated and highly agglomerated, indicating morphological changes in g-C_3_N_4_ induced by nickel doping.

**Figure 5:**
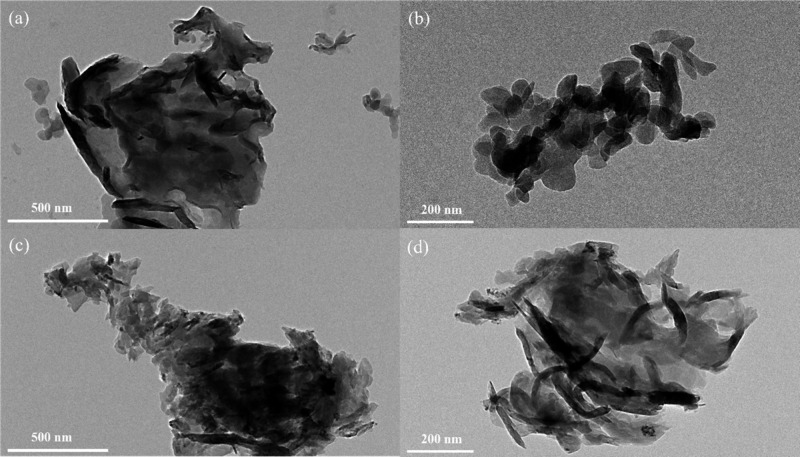
TEM images of (a, b) g-C_3_N_4_ and (c, d) Ni-*g*-C_3_N_4_.

### Effect of pH

The pH of the reaction medium plays a crucial role in the adsorption process, as it influences the charges of both the adsorbate (Dox) and the adsorbents (g-C_3_N_4_ and Ni-*g*-C_3_N_4_). The pH_pzc_, which indicates the pH at which the surface of the adsorbents is neutral, was found to be 6.42 for g-C_3_N_4_ and 6.50 for Ni-*g*-C_3_N_4_ (Figure 6(a)), consistent with the literature.^46^ When the pH of the solution is lower than the pH_pzc_, the adsorbent surfaces carry positive charges. In contrast, when the pH exceeds the pH_pzc_, the surfaces acquire negative charges.^47^

**Figure 6:**
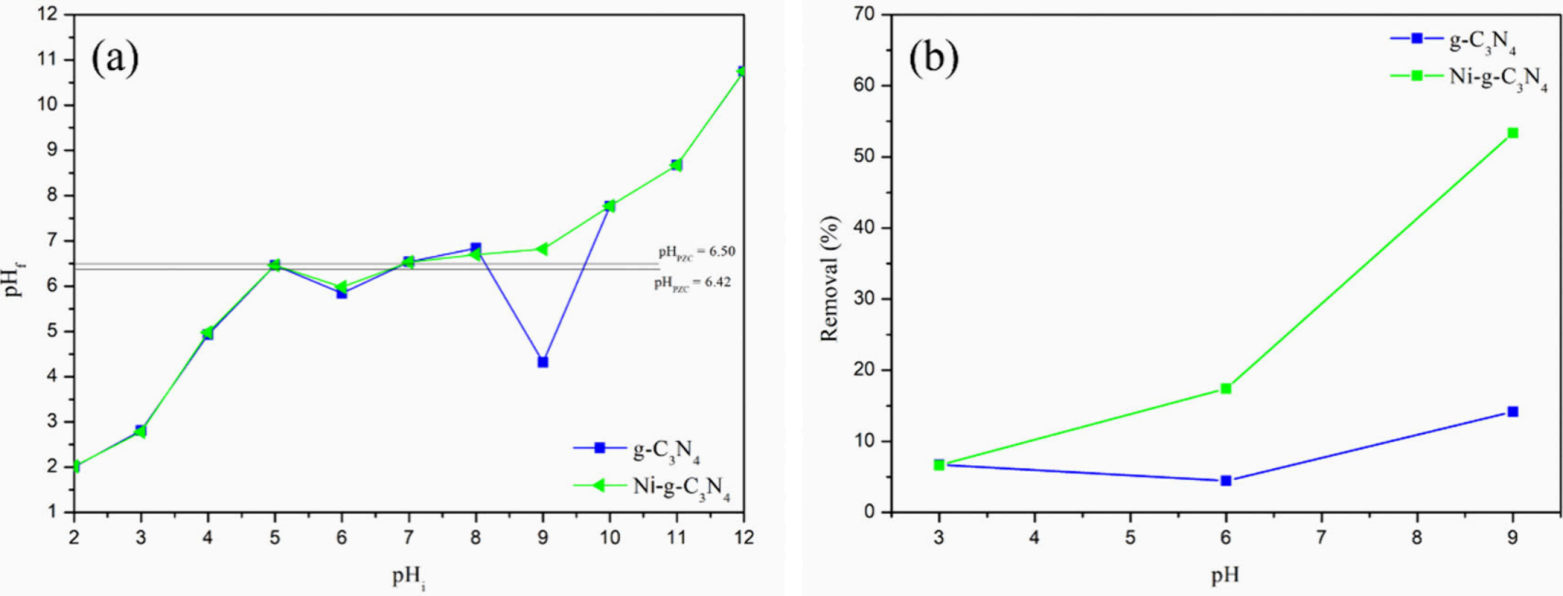
(a) Point zero charge (pH_pzc_) and (b) effect of pH on the Dox removal percentage for g-C_3_N_4_ and Ni-*g*-C_3_N_4_.

Tetracyclines, including Dox, are amphoteric molecules, meaning they contain three main ionizable groups in their chemical structure (alcohol, phenol, and amino). As a result, they can exist as cationic, anionic, or zwitterionic species, and their dissociation constants are influenced by the pH of the medium. At pH values below 3.02 (p*K*_a1_ = 3.02), Dox exists as a cationic molecule. Between pH 3.02 and 7.97 (p*K*_a2_ = 7.97), it exists as a zwitterion, and between pH 7.97 and 9.15 (p*K*_a3_ = 9.15), it is present as an anionic molecule. Above pH 9.15, Dox remains anionic due to the phenolic diketone moiety and tricarbonyl system losing protons.^48^

Figure 6(b) illustrates the impact of pH on the adsorption process of the samples. At pH 3, both g-C_3_N_4_ and Ni-*g*-C_3_N_4_ exhibit poor Dox adsorption, with only around 6.7% removal. At this pH, Dox is predominantly cationic, undergoing protonation due to the abundance of H^+^ ions in the solution. Similarly, the surfaces of both adsorbents are positively charged, leading to repulsion between the adsorbate and the adsorbent, which results in minimal adsorption.

At pH 6 and 9, Ni-*g*-C_3_N_4_ exhibited a significantly higher adsorptive capacity compared to pure g-C_3_N_4_. At pH 6, Dox is neutral, and both adsorbents are also neutral due to the pH of the medium being close to their respective pH_pzc_ values. In this condition, g-C_3_N_4_ and Ni-*g*-C_3_N_4_ removed 4.4% and 17.4% of Dox, respectively. At pH 9, the removal percentages for g-C_3_N_4_ and Ni-*g*-C_3_N_4_ increased to 14.2% and 53.4%, respectively. At this pH, Dox exists in its anionic form due to deprotonation at basic pH, and both adsorbents are negatively charged. The relatively low removal observed for g-C_3_N_4_ at pH 9 could be attributed to physisorption. For Ni-*g*-C_3_N_4_, the higher removal percentage can be ascribed to the presence of Ni^2+^ in the structure, which likely introduces positively charged regions. This enables stronger electrostatic interactions with the negatively charged Dox molecules, leading to more efficient adsorption. These changes in the properties of Ni-*g*-C_3_N_4_ will be further clarified by the results of the theoretical calculations. All subsequent experiments were conducted at pH 9, where both adsorbents demonstrated their best adsorptive capacity.

### Kinetic studies and adsorption isotherms

Figure 7(a) presents the removal percentage of Dox over different contact times for the adsorbents. For g-C_3_N_4_, only 25% of Dox was removed during the first 5 min of adsorption, which represented its maximum removal.^10^ Between 5 and 10 min, the removal percentage decreased by approximately 10%, and it remained relatively constant thereafter. In contrast, Ni-*g*-C_3_N_4_ exhibited a higher removal efficiency compared to the pure sample. Between 5 and 120 min, the removal percentage increased from 40% to 65%, respectively. This suggests that nickel doping enhanced the adsorption effectiveness of g-C_3_N_4_. After 120 min, the removal percentage stabilized, indicating that adsorption equilibrium had been reached. Furthermore, a slight decrease in the removal percentage was observed after 120 min, which could be attributed to electrostatic repulsion, because both Ni–g-C_3_N_4_ and DOX exhibited negative charges at the pH under study (pH 9).^49^ Although the highest removal efficiency was achieved at this pH, most likely due to the introduction of positively charged regions in the doped sample, the DOX molecule still exhibited negatively charged functional groups, which favored a partial desorption of the DOX after equilibrium was reached.

**Figure 7:**
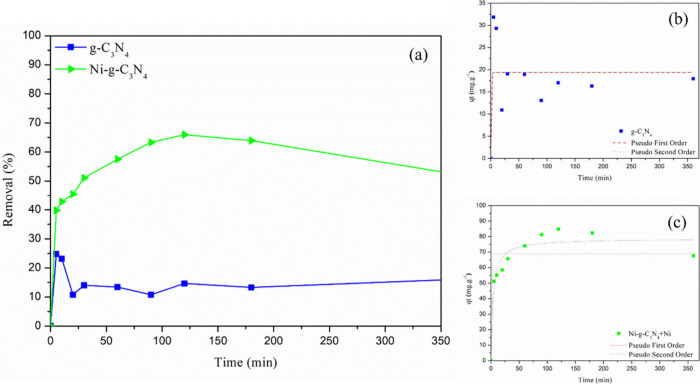
(a) Dox removal percentage of g-C_3_N_4_ and Ni-*g*-C_3_N_4_ over time. The adsorption kinetics of Dox fitted to the pseudo-first-order and pseudo-second-order kinetic models for (b) g-C_3_N_4_ and (c) Ni-*g*-C_3_N_4_.

The curves in Figure 7(b,c) illustrates the adsorption kinetics of Dox for the g-C_3_N_4_ and Ni-*g*-C_3_N_4_ adsorbents. The kinetic parameters of the adsorption process were calculated by fitting the data to the pseudo-first-order (eq 7) and pseudo-second-order (eq 8) models using linear regression.

7 $$q_{t}=q_{e}\cdot \left(1-\exp^{-k_{1}\cdot t}\right)$$

8 $$q_{t}=\left(k_{2}\cdot q_{e}^{2}\cdot t\right)/\left(1+\left(k_{2}\cdot q_{e}\cdot t\right)\right)$$

where *q_t_* (mg·g^–1^) and *q*_e_ (mg·g^–1^) are the adsorption capacities at time *t* (min) and at equilibrium, respectively; *k*_1_(min^–1^) is the pseudo-first-order rate constant; and *k*_2_ (g·mg ^–1^·min^–1^) is the pseudo-second-order rate constant.

Table 1 presents the adsorption kinetic parameters derived from the fitted models. For g-C_3_N_4_, the models did not provide a good fit, as observed in Figure 7(b) and the corresponding values in Table 1. In contrast, for Ni-*g*-C_3_N_4_, the data were best fitted to the pseudo-second-order model (Figure 7(c)), with a *k*_2_ value of 0.00345 and an *R* ^2^ of 0.926 (Table 1). The adsorption capacity *q*_e_ for Dox on Ni-*g*-C_3_N_4_ was found to be 78.644 mg·g^–1^. This suggests that the adsorptive process is primarily governed by the adsorbent’s ability to adsorb the contaminant.

**Table 1:** Adsorption Kinetic Parameters of Dox Fitted to the Pseudo-First-Order and Pseudo-Second-Order Kinetic Models for g-C_3_N_4_ and Ni-g-C_3_N_4_

	pseudo-first order			pseudo-second order		
adsorbents	*q*_e_ (mg·g^–1^)	*k*_1_ (min^–1^)	*R* ^2^	*q*_e_ (mg·g^–1^)	*k*_2_ (g·mg^–1^·min^–1^)	*R* ^2^
g-C_3_N_4_	19.385	444370.278	0.402	19.386	4.102 × 10^12^	0.402
Ni**-** *g* **-**C_3_N_4_	68.958	13.916	0.748	78.644	0.00345	0.926

To determine the maximum adsorptive capacity of Dox on both adsorbents, the Langmuir (eq 9), Freundlich (eq 10), and Sips (eq 11) models were applied in constructing the adsorption isotherms (Figure 8(a,b)).

9 $$q_{e}=q_{\max}\cdot \frac{\left(k_{L}.C_{e}\right)}{1+k_{L}.C_{e}}$$

10 $$q_{e}=k_{F}\left(C_{e}^{1/n}\right))$$

11 $$q_{e}=q_{\max}\left({\left(k\cdot C_{e}\right)}^{n}\right)/\left({\left(1+k_{S}\cdot C_{e}\right)}^{n}\right)$$

where *q*_e_ (mg·g^–1^) and *q*_max_ (mg·g^–1^) are the adsorption capacity at equilibrium and maximum adsorption capacity, respectively; *C*_e_ (mg·L^–1^) is the concentration of Dox at equilibrium; *K*_L_ (L·mg^–1^) is the Langmuir constant; *k*_F_ (mg·g^–1^) and *n* are constants, i.e., adsorption capacity and adsorption intensity factor, respectively; and *k*_S_ (L·mg^–1^) is the adsorption constant of the Sips model.

**Figure 8:**
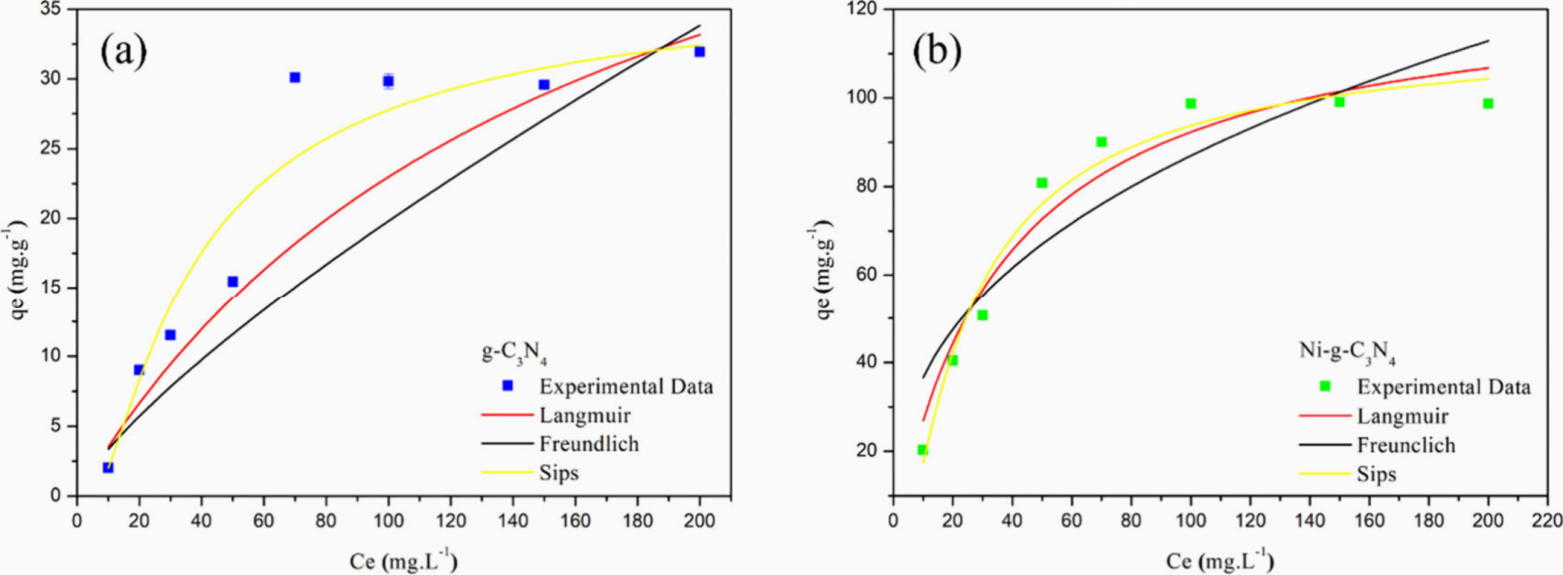
Adsorption isotherms of Dox for (a) g-C_3_N_4_ and (b) Ni-*g*-C_3_N_4_.

Table 2 presents the parameters obtained from each adsorption model. All the evaluated models showed good adherence, but the best fit was observed for the Sips model. The Sips model, being a combination of the Langmuir and Freundlich models, is more suitable for describing heterogeneous adsorption systems. The maximum adsorption capacity (*q*_max_) of Ni-*g*-C_3_N_4_ was 116.265 mg·g^–1^, which is at least three times higher than that of g-C_3_N_4_ (37.889 mg·g^–1^). Bystrzejewski et al. (2017)^16^ synthesized Na-doped g-C_3_N_4_ and evaluated its adsorption performance for the removal of the methylene blue dye. XRD analysis confirmed that the samples prepared with up to 10% NaCl did not exhibit additional phase formation, and the sample containing 10% NaCl exhibited the highest adsorption capacity (275 mg·g^–1^) compared with 10 mg·g^–1^ for g-C_3_N_4_. However, these studies are solely focused on dye removal, with no reported investigations into the adsorption of pharmaceuticals using doped g-C_3_N_4_.

**Table 2:** Parameters Obtained from Langmuir, Freundlich, and Sips Isotherms for Dox Adsorption by Pure g-C_3_N_4_ and Ni-g-C_3_N_4_

	models								
	Langmuir		Freundlich			Sips			
adsorbents	*q*_m__a__x_ (mg·g^–1^)	*k*_L_ (L·mg^–1^)	*R* ^2^	*n*	*k*_f_ (mg·g^–1^)	*R* ^2^	*q*_m__a__x_ (mg·g^–1^)	*k*_s_ (L·mg^–1^)	*R* ^2^
g**-**C_3_N_4_	59.632	0.006	0.925	1.294	0.564	0.889	37.889	0.791	0.976
Ni**-**g**-**C_3_N_4_	126.499	0.027	0.943	2.660	15.639	0.814	116.265	0.311	0.965

These results indicate that doping g-C_3_N_4_ with nickel significantly enhanced its adsorption capacity, improving the removal of the contaminant. The adsorptive process for both pure g-C_3_N_4_ and Ni-*g*-C_3_N_4_ was further studied in the following theoretical calculations.

### Computer Simulations

#### Molecular of Electrostatic Potential and Frontier Molecular Orbitals

MEP provides valuable insights into the chemical reactivity of molecules and their electron density distribution on a surface. It serves as a tool for identifying potential reaction sites.^50,51^ MEP is represented in color, with blue indicating a positive potential (electron-deficient regions that tend to attract nucleophiles), green or white indicating neutral potential, and red representing a negative potential (electron-rich regions that may attract electrophiles).^50,51^

FMO analysis is critical in chemistry for evaluating chemical reactions, predicting molecular interactions, and identifying reactive sites, which explains its widespread application across various chemical studies.^52^

In the context of this study, the FMOs and MEP were analyzed for the g-C_3_N_4_ and Ni-g-C_3_N_4_ adsorbents, as shown in Figure 9.

**Figure 9:**
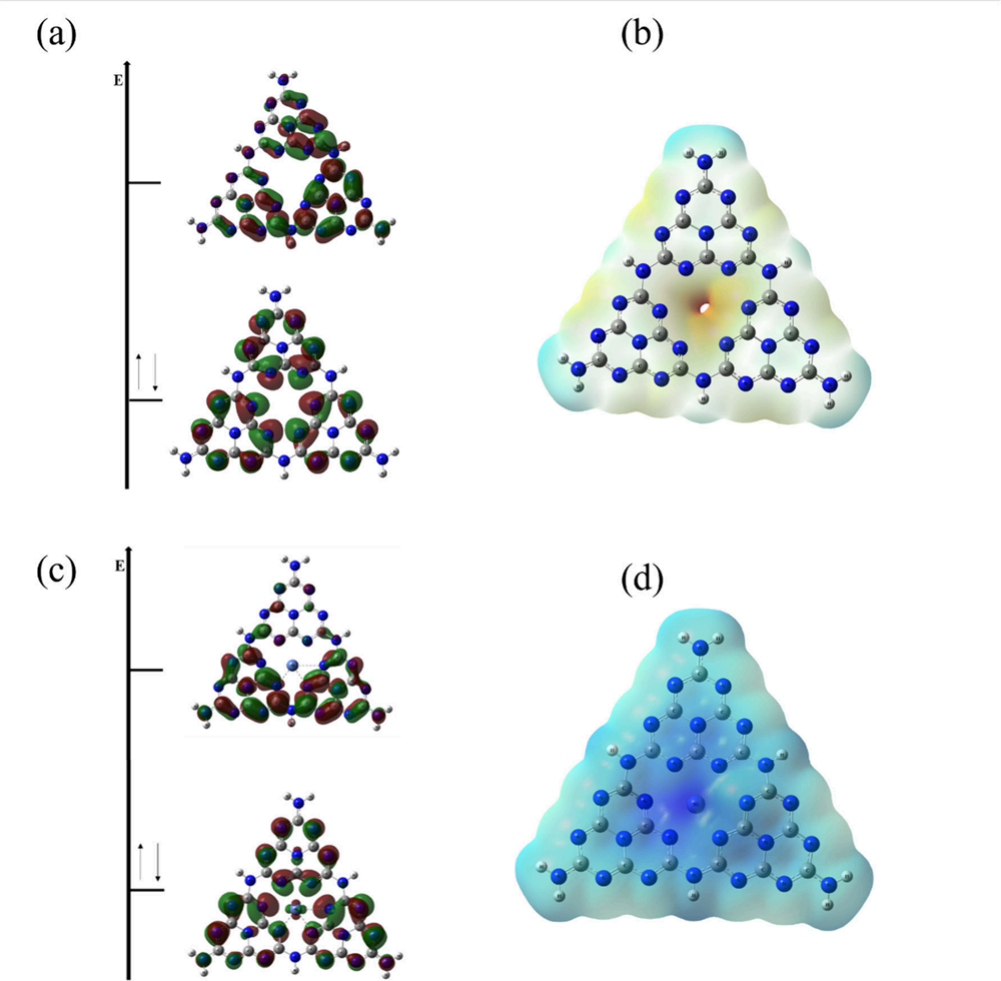
HOMO–LUMO and MEP for (a, b) g-C_3_N_4_ and (c, d) Ni-g-C_3_N_4_.

FMOs of g-C_3_N_4_ reveal well-defined π orbitals due to the presence of nitrogen and aromatic ring electrons (Figure 9(a)). MEP displays a reddish color at the center of the molecule, indicating regions rich in electrons, while the surrounding areas, primarily consisting of hydrogens, show a bluish distribution (Figure 9(b)). The doping of g-C_3_N_4_ with Ni occurs at the center of the structure. The optimized structure reveals that the FMOs maintain well-defined π orbitals, with the probability density of HOMO distributed across the entire molecule. In contrast, the LUMO exhibits a probability density concentrated at the lower part of the molecule (Figure 9(c)). The MEP of Ni-g-C_3_N_4_ (Figure 9(d)) shows a bluish color, indicating the presence of Ni^2+^ in the structure.

This positive MEP correlates with the enhanced adsorption capacity observed experimentally, particularly at alkaline pH (9), where the positive potential of the sample tends to attract negatively charged molecules. The structural parameters of the Ni-g-C_3_N_4_ are shown in Figure 10.

**Figure 10:**
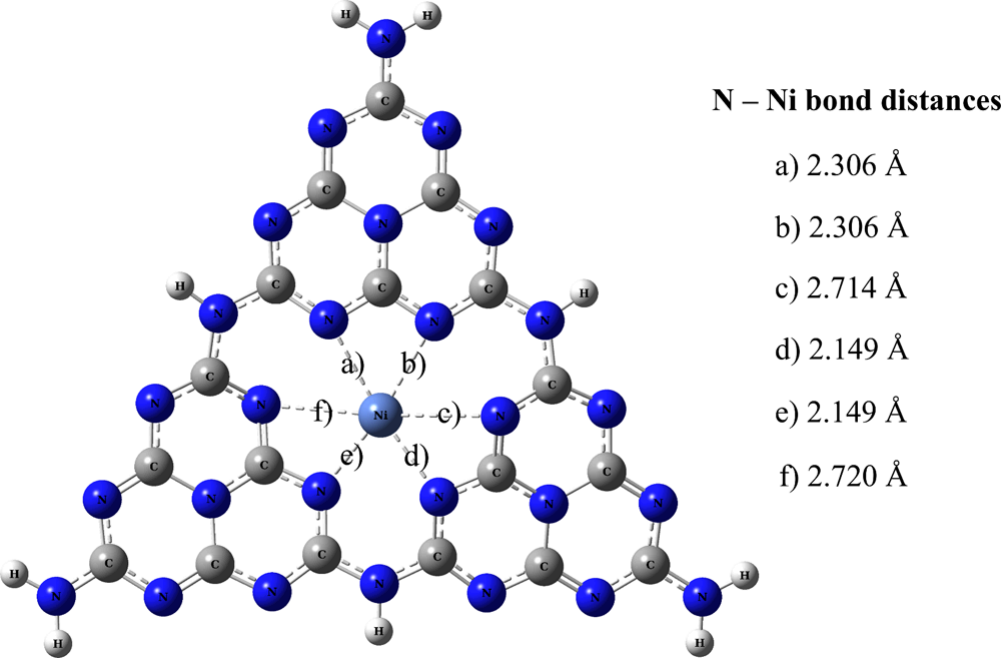
Representation of bonds resulting from nickel doping in g-C_3_N_4_ (Ni-g-C_3_N_4_).

Figure 10 shows that the N–Ni bond lengths in the Ni-g-C_3_N_4_ structure range from 2.306 to 2.720 Å, with six N–Ni bonds formed. This confirms that Ni^2+^ is effectively integrated into the central structure of g-C_3_N_4_.

In addition to the adsorbents, the adsorbate, Dox, was also examined theoretically. Three different forms of Dox were analyzed: the neutral Dox (Figure 11(a,b)), Dox with one deprotonation at the −NH_2_ group (Figure 11(c,d)), and Dox with two deprotonations, one at the NH_2_ and the other at the OH group (Figure 11(d,e)). The FMO and MEP simulations for these forms were carried out considering deprotonation because the experimental results showed the best performance at basic pH.

**Figure 11:**
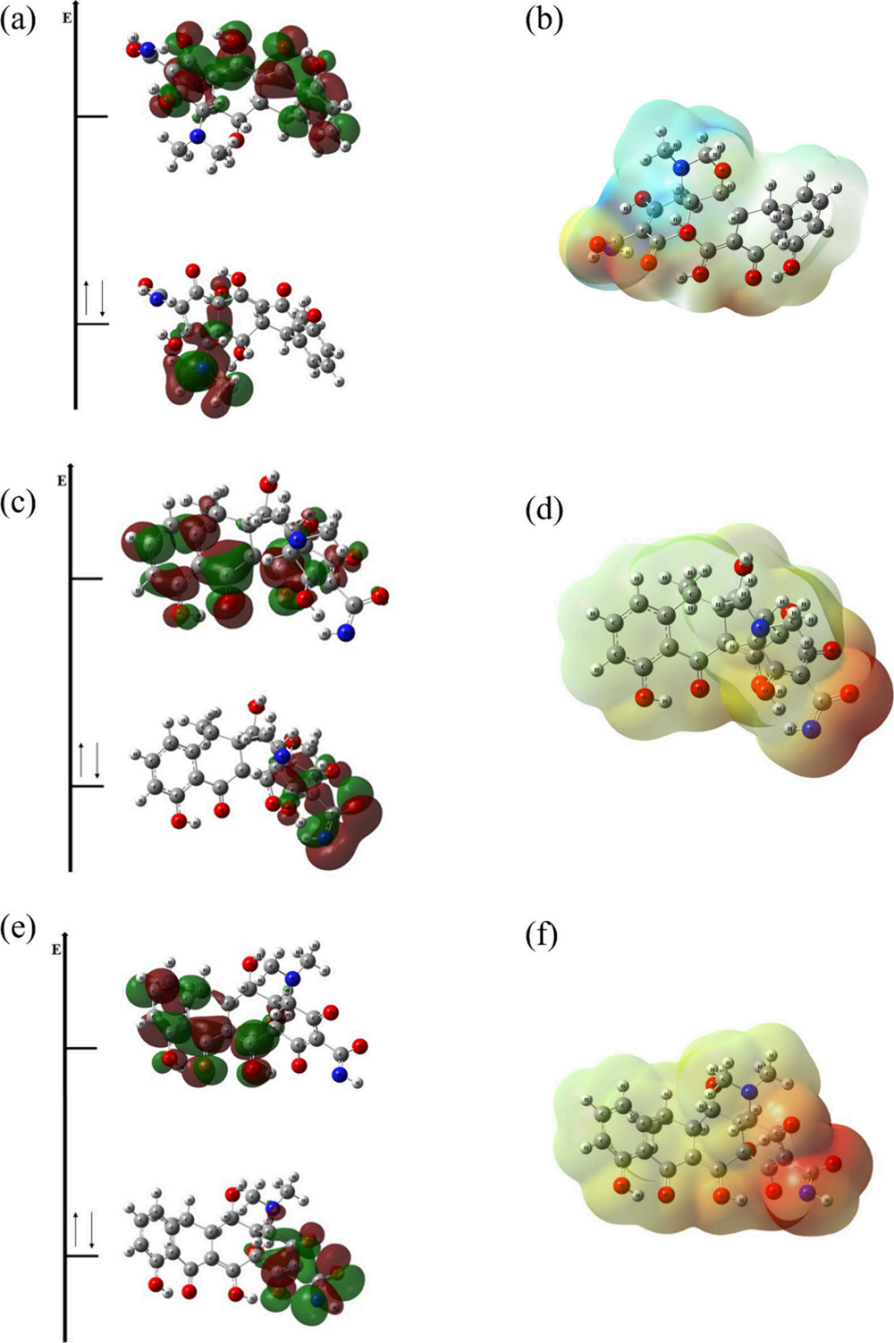
(a, b) HOMO–LUMO and MEP of neutral Dox; (c, d) deprotonated Dox in the NH_2_ group; (e, f) Dox with two deprotonations, one in the NH_2_ group and the other in the OH group.

For neutral Dox (Figure 11(a)), the FMOs show that the HOMO is located on the – N–(CH_3_)_2_ group, while the LUMO resides on the aromatic rings of the structure. The MEP shown in Figure 11(b) indicates negative charge density on the oxygen and nitrogen atoms of the molecule, with a partially positive charge on the remaining parts of the molecule. For the deprotonated forms of Dox (Figure 11(d,f)), the MEP reveals a reddish color, reflecting the negative charge resulting from the deprotonation. When two deprotonations occur, the intensity of charge density within the molecule increases.

The FMOs of these deprotonated molecules show that the LUMO is primarily located on the aromatic ring, with some extension to the attached ring (Figure 11(c,e)). The HOMO is found on the group where deprotonation occurred: on the −NH– group for a single deprotonation (Figure 11(c)) and on both the – NH^–^ and O^–^ groups when two deprotonations take place (Figure 11(e)).

#### Binding Energy, Enthalpy, and Gibbs Free Energy

To evaluate the magnitude of the interactions between the adsorbents and adsorbates, the electronic interaction energy (Δ*E*_int_), enthalpy (Δ*H*), and Gibbs free energy (Δ*G*) were calculated, as shown in Table 3.

**Table 3:** Electronic Interaction Energies (Δ*E*_int_) at 0 K, Enthalpy Variation (Δ_r_ *Η*), and Gibbs Free Energy (Δ_r_ *G*) at 298 K for the Studied Complexes^a^

complexes	Δ*E*_int_	Δ_r_ *H*	Δ_r_ *G*
g-C_3_N_4_.···· Dox	–17.50	–17.49	–0.21
g-C_3_N_4._···· Dox _deprotonated_	–16.28	–16.63	2.80
Ni-g-C_3_N_4_.···· Dox _deprotonated_	–56.94	–57.07	–40.95
Ni-g-C_3_N_4_.···· Dox _2 deprotonations_	–61.59	–61.82	–44.22

a Data are shown in kcal mol^–1^.

The interactions were studied for different configurations based on both experimental data and theoretical calculations, including the analysis of FMOs and MEPs. The following interaction scenarios were considered: (i) interaction of g-C_3_N_4_ with neutral Dox molecules (g-C_3_N_4_·····Dox), (ii) interaction of g-C_3_N_4_ with deprotonated Dox molecules at the NH_2_ group (g-C_3_N_4_·····Dox_Deprotonated_), (iii) interaction of Ni-g-C_3_N_4_ with deprotonated Dox molecules at the NH_2_ group (Ni-g-C_3_N_4_·····Dox _deprotonated_), and (iv) interaction of Ni-g-C_3_N_4_ with Dox molecules exhibiting two deprotonations—one at the – NH_2_ group and the other at the – OH group (Ni-g-C_3_N_4_·····Dox_2deprotonations_) (Table 3).

Based on the ΔE_int_ results presented in Table 3, it is clear that the g-C_3_N_4_ matrix shows a tendency to interact with both neutral and deprotonated Dox molecules. However, the positive ΔG values suggest that these interactions are nonspontaneous. Additionally, the Δ*E*_int_ values are relatively low, which points to weak interactions. These findings align with the experimental results shown in Figure 8(a), where pure g-C_3_N_4_ exhibited desorption after the first 5 min, indicating physical adsorption rather than a chemical bond.

For the Ni-g-C_3_N_4_ sample, the MEP analysis reveals a positive charge density due to the presence Ni^2+^ ions in the structure, which enhances its interaction with negatively charged species. Therefore, only interactions with the deprotonated Dox molecule were considered. The results for Ni-g-C_3_N_4_ indicate significantly better adsorption performance, as reflected by the ΔE_int_ values below 0.00 kcal mol^–1^, indicating favorable interactions. The ΔH values for these interactions indicate heat release, supporting the notion that the adsorption process is exothermic. Moreover, all evaluated scenarios for Ni-g-C_3_N_4_ show spontaneous adsorption (Δ*G* < 0.00 kcal mol^–1^), consistent with a chemical process. The isotherm data in Figure 8(b) further supports these theoretical findings. Ni-g-C_3_N_4_ demonstrates a higher adsorption capacity for Dox compared to pure g-C_3_N_4_, with no desorption observed, reinforcing the conclusion that the interaction in Ni-g-C_3_N_4_ is chemical rather than physical.

## Conclusions

In conclusion, both g-C_3_N_4_ and Ni-g-C_3_N_4_ samples were successfully synthesized through the simple thermal polycondensation method, as confirmed by XRD, FTIR, and XPS analyses. These analyses indicated that Ni^2+^ ions were incorporated into the g-C_3_N_4_ structure without the formation of additional phases, with Ni^2+^ evenly distributed throughout the sample. While doping did not significantly alter the overall structure of g-C_3_N_4_, the morphology of Ni-g-C_3_N_4_ was slightly modified, as observed through morphological analyses. The Ni-g-C_3_N_4_ adsorbent demonstrated superior adsorptive capacity compared to pure g-C_3_N_4_, highlighting that nickel doping enhanced the material’s ability to adsorb ECs, specifically Dox in this study. Both adsorbents showed improved Dox removal at an alkaline pH of 9, where the drug was deprotonated and existed in its anionic form. The g-C_3_N_4_ sample exhibited a low removal rate (14.2%), primarily due to physisorption, as confirmed by thermodynamic parameters from theoretical calculations. In contrast, Ni-g-C_3_N_4_ achieved a higher removal rate (53.4%), attributed to the Ni^2+^ ions, which modified the surface charge of g-C_3_N_4_, thereby promoting a more efficient adsorptive process. The theoretical simulations revealed that nickel doping of g-C_3_N_4_ significantly influenced the adsorption mechanism, shifting the process from physisorption to chemisorption. This was further supported by thermodynamic and bonding parameter analysis, demonstrating the effectiveness of Ni-g-C_3_N_4_ in adsorptive applications. Thus, the study underscores the potential of Ni-g-C_3_N_4_ as a promising material for efficient adsorption of contaminants, with the doping process playing a crucial role in enhancing the material’s adsorptive properties.
